# The Inspection against Small-Pox on the Canadian Frontier

**Published:** 1885-11

**Authors:** 


### Editorial.

---

### The Inspection against Small-Pox on the Canadian Frontier.

Since the issue of our last number, small-pox has become more virulent than ever in Montreal. The number of deaths for the week ending October 18th exceeded that of any previous week, while the disease has, at the same time, been extending. Cases have been reported in Quebec, Three Rivers, Hamilton and St. Catherines, the contagion making a slow but steady march toward the American frontier.

While the other frontier States took active measures to prevent the entrance of the contagion within their borders, New York was doing nothing, even when there were cases of small-pox within a dozen miles of her boundary line. The city of Buffalo, which had already had some sad experience with this pestilence, was especially anxious, and called upon the State Board of Health for assistance. This was refused, on the ground that the appropriation for the prevention of epidemics had been vetoed. The municipal Board of Health then asked the national government for aid, and received the reply that such aid would be granted when the Governor of New York requested it. In the midst of this uncertainty, the local Board of Health of the city of Buffalo instituted, of its own accord, a quarantine against the infected districts of Canada. The railroad authorities and the Canadian government co-operated with the board in a very commendable manner, and, on the 7th of October, inspectors were appointed, who examined the passengers and baggage on all trains crossing the Niagara river from Canada. While these measures were originally intended only to protect the city of Buffalo, the system of inspection established has really protected the entire State of New York. The great majority of the passengers coming from Canada carry certificates of recent vaccination, and all others coming from towns where small-pox existed were obliged to submit to this operation.

In the meantime, government aid had been granted, and, on the 22d of October, Surgeon A. W. Austin, of the United States Marine Hospital Service, appointed inspectors and assumed entire charge of the quarantine. The following instructions have been issued to the inspectors:

1. All persons bound for the United States coming from Montreal, or other places in Canada where small-pox prevails, must produce satisfactory evidence to the inspector that they are protected by a recent vaccination, or submit to this operation before being allowed to cross the boundary line.

2. Inspectors will vaccinate all unprotected persons free of charge.

3. Persons coming from Montreal or suburban villages will be carefully questioned as to their residence, whether small-pox has occurred in their families, or whether they have been in contact with the disease.

4. Inquiries should also be made relative to their baggage, whether it consists of bedding, household goods, etc., likely to be infected, and if any person or article of baggage is considered by the inspector infected, or likely to introduce the disease into the country, they or it should not be allowed to cross the line into the United States.

5. You may consider persons protected who may show evidences of having had the small-pox or varioloid, or who exhibit a well-defined mark of vaccination.

6. You may accept as evidence of protection a certificate from any physician in good standing, that the person presenting the same has been recently vaccinated. Should you doubt the validity or authenticity of the certificate, you may refuse any person presenting the same the privilege of crossing the border unless he will submit to vaccination. Baggage known to have come from any infected district and believed to be infected, will be thoroughly fumigated with burning sulphur.

We cannot close this subject without speaking a word in commendation of our efficient Health Physician, Dr. A. H. Briggs. He is the very soul of the Board of Health, being the first to perceive any danger to the public health, and his activity is untiring until the danger is removed or averted. It was by his advice that the inspection against small-pox was organized. It was by his advice that a system of sanitary inspection of private property was instituted; and, by his zeal, he has constructed a health department for Buffalo which, as Dr. E. M. Moore, president of the New York State Board of Health, has remarked, is unsurpassed in efficiency by that of any city in the United States.

---

Acting under the provisions of the Act "Regulating and restraining the Practice of Midwifery in Erie county," in July last Judge Hammond appointed, as a Board of Examiners, Drs. M. D. Mann, H. R. Hopkins, P. W. Van Peyma, J. H. Pryor and J. W. Keene. The board held its first meeting Oct. 6, 1885. They have examined about 38 applicants up to date. About 60 per cent. of those examined passed and were admitted to practice in this county. It is hoped this will lead to much good, and correct many abuses which existed before. This is the first law of this kind in operation in this State, and the first in the United States to take such decided steps in the regulation of the practice of midwifery. The physicians who so ably presented this subject and skillfully managed to get the endorsement of the profession of this county against decided opposition, are deserving of great credit. For the benefit of our readers in other counties and States, we append a copy of this bill, with the hope that those who need it will be successful in obtaining one this year. The text of the Act is as follows:

"SECTION 1. On or before the 1st day of July, 1885, the County Judge of Erie county shall, by an order to be filed in the Erie County Clerk's office, appoint a board of examiners in midwifery, to consist of five members, who shall have been licensed to practice physic and surgery in this State, and thereafter, as often as any vacancy shall occur in said board, said county judge shall, by a like order, fill such vacancy.

"SEC. 2. Immediately after the filing of said order, said board shall organize, by the selection of one of its members as president, and of another as secretary and treasurer, who shall hold their office for one year, and be thereafter annually elected, and shall adopt and have power to adopt and enforce such rules and regulations as are necessary to carry out the purposes and provisions of this Act.

"SEC. 3. Such examiners shall meet on the first Tuesdays of October and April in each year, and on such other days as such board may appoint, in the city of Buffalo, after due notice thereof is publicly given, and shall then examine all candidates of the age of 21 years and upwards, possessed of good moral character, who shall present themselves to be examined for license to practice midwifery in the county of Erie, and shall, on receipt of $10, issue their certificate to any person so examined who shall be found by them to be qualified, which certificate shall set forth that said board has found the person to whom it is issued, qualified to practice midwifery, and shall be recorded by the Clerk of the county of Erie in a book to be kept by him for that purpose. All moneys going into the treasury of this board shall be applied to defray the expenses of this board.

"SEC. 4. Any person who has received and recorded such certificate, shall thereupon be designated a midwife, and authorized and entitled within the county of Erie to practice midwifery in cases of normal labor and in no others; but such persons shall not in any case of labor use instruments of any kind, nor assist labor by any artificial, forcible or mechanical means, nor perform any version nor attempt to remove adherent placenta, nor administer, prescribe, advise or employ any poisonous or dangerous drug, herb or medicine, nor attempt the treatment of disease except where the attendance of a physician cannot be speedily procured, and in such cases such person shall at once, and in the most speedy way, procure the attendance of a physician.

"SEC. 5. Said board of examiners shall have power, on proper cause shown, and after hearing the person holding their certificate, to recommend to the County Judge of Erie county the revocation of the same, and said Judge shall have power to revoke such certificate and license.

"SEC. 6. Any person who shall practice or, without the attendance of a physician where one can be procured, attend a case of midwifery or obstetrics within the county of Erie, after the 31st day of December, 1885, without being duly authorized so to do under existing laws of this State, or without having received and recorded the certificate provided for by this Act, and any person who shall violate any of the provisions of this Act, shall be guilty of a misdemeanor, and, on conviction thereof, shall be fined not less than $50 nor more than $100, and shall forfeit any certificate heretofore granted under the provision of this Act."

---

The New York State Medical Association meets at the Murray Hill Hotel, in New York City, on November 17, 18 and 19, 1885. This association has now a large membership, and it is fast increasing in numbers and influence. The meeting last year was one of the most interesting and instructive gatherings of this kind ever held. The programme was unusually well carried out.

This year, as last, the programme is as good as could be well arranged, and everything points to a very interesting and successful meeting. Some of the subjects are to be presented by men who can speak with authority. Dr. A. L. Carroll, Secretary of the State Board of Health, will present an address on "State Medicine." Prof. Austin Flint will present a paper in which he propounds eight questions relative to the "Nature, pathology, prognosis and treatment of acute lobar pneumonia, each question to be answered by two or more Fellows." Prof. A. Flint, Jr., will present an address on "Some of the Relations of Physiology to the Practice of Medicine." Prof. Edward Janeway will give an address on "Pathology." Prof. Lewis H. Sayre, one on "The Treatment of Spondylitis and treatment of caries of the spine by partial suspension and the plaster jacket, and the treatment of lateral curvature by gymnastics and partial suspension and the plaster of Paris corset. Demonstrations of practical applications to cases." "Notes on contracture of the bladder consequent upon cystitis," by Prof. John W. S. Gouley, M. D.

Among those who are to participate from this county are Prof. Thos. F. Rochester, who takes part in the discussion on "Pneumonia," and Prof. Charles G. Stockton, on the same subject. Prof. W. S. Tremaine will read on "Tumors of the Jaw." Dr. U. C. Lynde will present two papers, "Skin Flaps in Amputations" and "Umbilical Hemorrhages in Infants." A paper on "Nutrition in Lithæmia," by Chas. G. Stockton, M.D., will be read; "The proper disposition of street sweepings, ashes, swill and garbage in our cities," by Albert H. Briggs, M.D.

Why this association was formed we all know; its object is a worthy one, and it should be sustained by every member of the Erie County Medical Society. If only on this account, every one should attend this meeting, and to those who attend, it will be a source of great profit.

---

The vacancy created by the resignation of Dr. Floyd S. Crego, as Second Assistant Physician of the Buffalo State Asylum, last June, has just been filled. From among many applicants, Dr. A. W. Hurd, of New York, has been selected, and was appointed by the board, at their last meeting, held October 14th. He was graduated with honors at the College of Physicians and Surgeons of New York, and served two years in the hospitals of that city. Subsequently he studied in Germany. All of this constitutes preparation which should make him a valuable addition to the medical staff of this institution. The Doctor passed the civil service examination in a most creditable manner, having received 91.89 per cent. When we consider that this examination includes many subjects besides strictly medical topics, it is all the more worthy of credit. The method of filling this appointment is more satisfactory than by a competitive examination. In this case, it was confined to the person found to possess the necessary qualifications, and, on this account, was recommended to the Commission. This obviates the danger which exists in a competitive examination of putting a man in a certain office, who, outside of professional qualifications, might be entirely unfit for the position. Dr. Hurd has been at the Asylum for some weeks, and we learn from a reliable source that he proves himself not only fully competent to perform the duties, but that he possesses the qualities of heart which endear him to the patients and inspire confidence in all.

The civil service, as applied to asylums, does not correct any abuse; for no officer nor physician, in the past, has obtained place in them through political influence, but it assures to these institutions perfect freedom from this baneful influence in the future.

---

Dr. C. C. F. Gay, of this city, returned last month from a trip to Europe, having been absent several months. The Doctor went in search of health, and our readers will be delighted to hear he has completely regained it. We have received two letters from him, which contain many points of interest, and we are greatly indebted to him for them. The first one we published in our September number, and the second we print in this issue. Although the Doctor went in search of health, he could not neglect the opportunity to meet medical men in other lands and get new ideas for the use of the profession and humanity. The members of the faculty of Niagara University, on the day of his arrival, tendered him a reception. About fifty members of the profession were present, by invitation, to meet him. After the formal reception, at which Rt. Rev. Bishop Ryan and Prof. Cronyn received, the gentlemen present sat down to enjoy a repast. Prof. Cronyn gave a short address of welcome, and Dr. Gay responded in a few well-chosen remarks. It was a very enjoyable evening, and those present wished the Doctor long life and health to continue his professional labors, which in the past have been performed with such zeal and devotion to the rich and poor alike.

